# Clinical Experience with Hemopatch® as a Dural Sealant in Cranial Neurosurgery

**DOI:** 10.7759/cureus.4013

**Published:** 2019-02-04

**Authors:** Karl-Michael Schebesch, Alexander Brawanski

**Affiliations:** 1 Department of Neurosurgery, University Medical Centre of Regensburg, Regensburg, DEU

**Keywords:** dura substitute, hemopatch, dural sealant

## Abstract

Background: Herein, we report our clinical experience with the novel polyethylene glycol-covered matrix dural onlay, Hemopatch® (Baxter Deutschland GmbH, Unterschleißheim, Germany) for the prevention of postoperative cerebrospinal fluid (CSF) fistulas.

Methods: Retrospectively, 22 consecutive patients (11 females, 11 males, mean age: 49.8 years, range: 15–77 years) with oncological and vascular intracranial lesions were included in this study. In all patients, the Hemopatch was applied as the dural onlay. The accuracy of the primary dural sutures was distinguished into 1) no visible gaps, 2) small gaps < 3 mm, and 3) large gaps > 3 mm. We evaluated the patient charts, surgical reports, and postoperative images. The median follow-up was three months. We recorded any wound healing disorder, such as infection or CSF fistula, and postoperative hemorrhage resulting in surgical revision.

Results: Supratentorial, infratentorial, and transsphenoidal approaches were conducted in 17, four, and one patient, respectively. Accurate sutures without visible gaps, small gaps, and large gaps were covered with the Hemopatch in 11, eight, and three patients. One patient developed a CSF fistula (4.5%), one patient had a wound infection (4.5%), and in one patient, a remote cerebellar hemorrhage occurred (unrelated to the dural closure) (4.5%). Thus, the surgical revision rate due to wound healing disorders was 9% (2/22).

Conclusion: It is safe and feasible to use the Hemopatch as a dural sealant. The rate of postoperative wound healing disorders in our population was in the lower range of reported surgical revision rates after supra-/infratentorial craniotomies. However, prospective and controlled clinical trials are still warranted.

## Introduction

The reported rate of cerebrospinal fluid (CSF) leakage after craniotomy ranges widely from 4% - 32% [1-2] and, specifically, up to 17% after infratentorial approaches [3-4]. CSF fistulas are not the only significant contributors to a surgical site infection [5]. CSF leakages are among the most frequently reported indicators for surgical revisions, frequently delaying the start of adjuvant treatment and early rehabilitation in neuro-oncological diseases or after brain trauma. Furthermore, the annual socioeconomic burden is high due to extensive re-treatment of the CSF-fistula [6].

A watertight dural closure can be achieved by direct suturing of the dural rims; eventually, an autologous graft-like muscle flap or periosteum is placed on visible gaps and fixed to the dura by additional sutures or with fibrin glue. However, in many cases, it appears safer and more comfortable to cover obvious gaps with adherent collagenous layers. A variety of artificial sealants of this type are available; some are suturable and others are simple onlays, pressed on the dural matrix with wet or dry cotton.

The novel dural onlay, Hemopatch® (Baxter Deutschland GmbH, Unterschleißheim, Germany), has only been released recently [7]. It is a polyethylene glycol-covered matrix, derived from the bovine dermis, which has been proven to be an effective sealant in animal models and in clinical practice [8-11]. Herein, we present our first clinical experiences in different intracranial, supra-/infratentorial procedures with the Hemopatch as a dural sealant. We focused on the rate of wound healing disorders, such as CSF leakages and wound infections, within 12 weeks after surgery.

## Materials and methods

**Figure 1:**
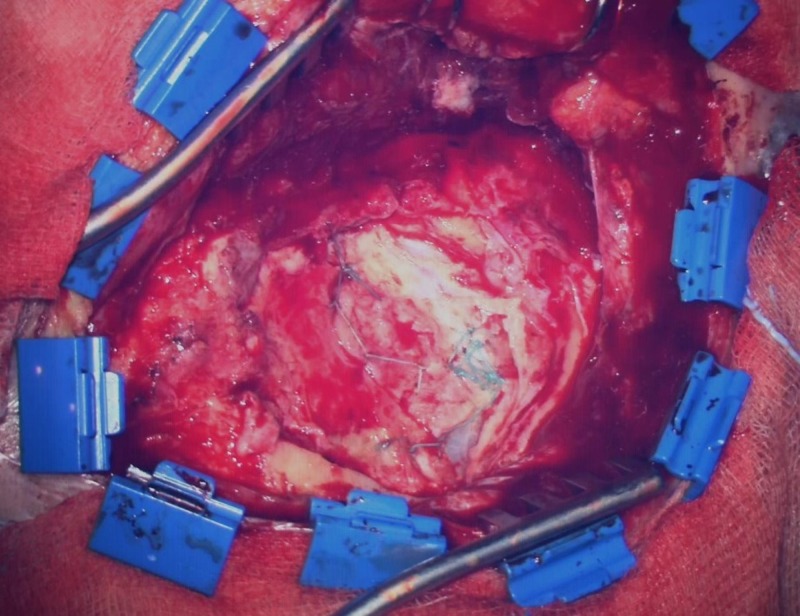
Accurate suturing of the dural rims (no visible gaps)

**Figure 2:**
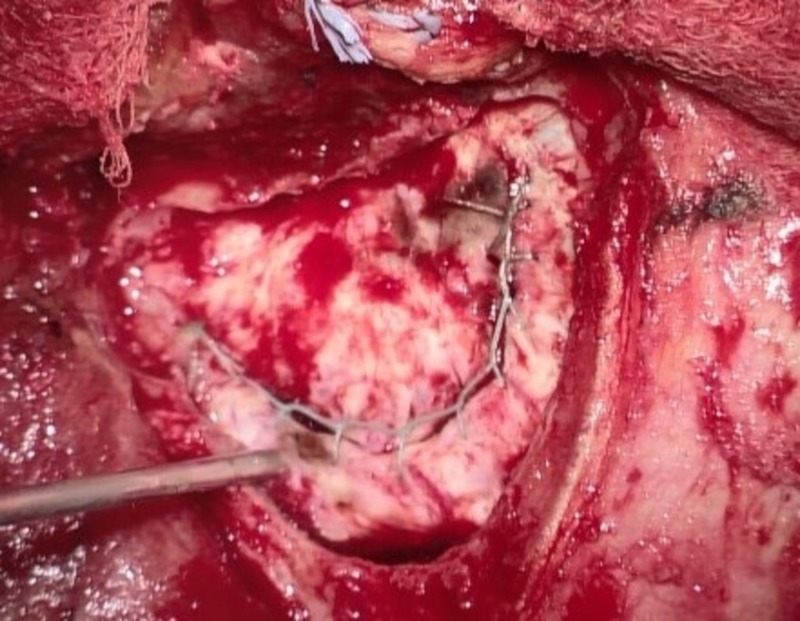
Small gaps after dural suturing (< 3 mm)

**Figure 3:**
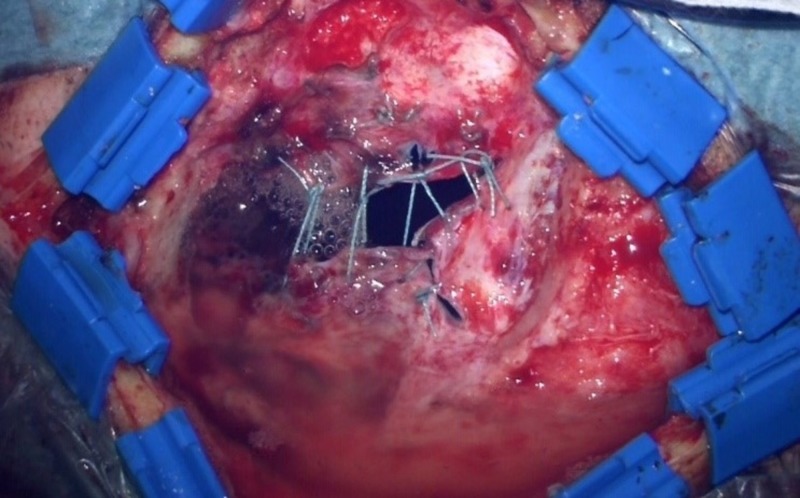
Large gaps after dural suturing (> 3 mm)

We retrospectively analyzed 22 consecutive patients (11 females, 11 males, mean age: 49.8 years, range: 15 -77 years) in whom the Hemopatch was used as the only artificial dural add-on after the dural rims had been adapted by suture as tightly as possible. All patients were treated surgically for the first time (no reoperation), no fibrin glue, and no other additional artificial material was used. According to our standard, all patients received a single-shot intravenous antibiotic before skin incision. The bone flap was fixed with plates and mini-screws in all cases. All patients were operated on by one responsible neurosurgeon (KMS). The charts and surgical reports were screened and the postoperative scans, computed tomography (CT) or magnetic resonance imaging (MRI), were evaluated. On the basis of the surgical reports and according to the intraoperative video captures and photographs (conducted in 20 patients), a distinction was made between no visible gaps between dural rims (Figure 1), small dural gaps < 3 mm (Figure 2), and large dural gaps > 3 mm (Figure 3). The Institutional Review Board of the University of Regensburg approved this study (#18-1177-104).

**Figure 4:**
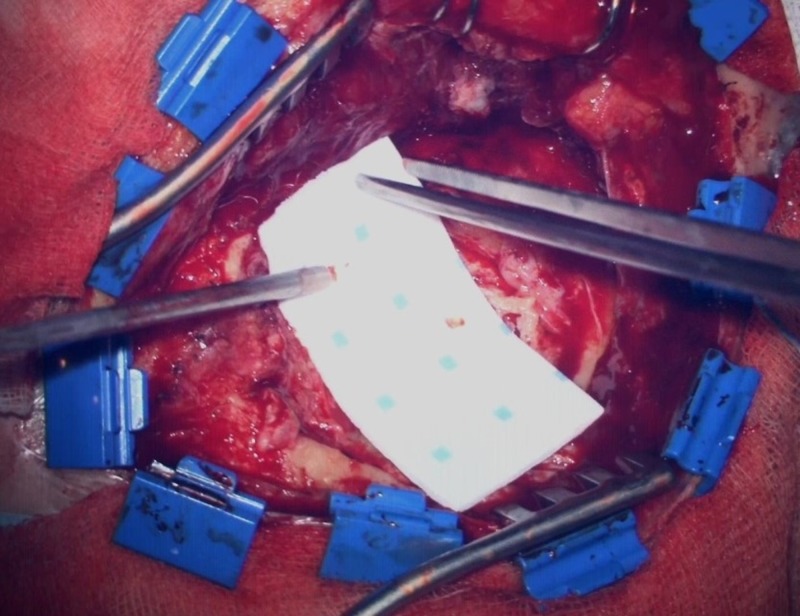
Additional covering with the Hemopatch

**Figure 5:**
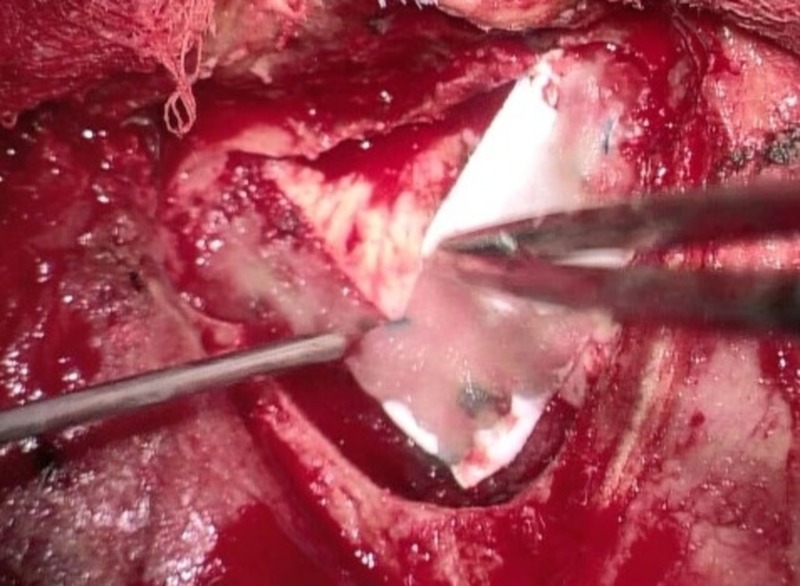
Covered with the Hemopatch

**Figure 6:**
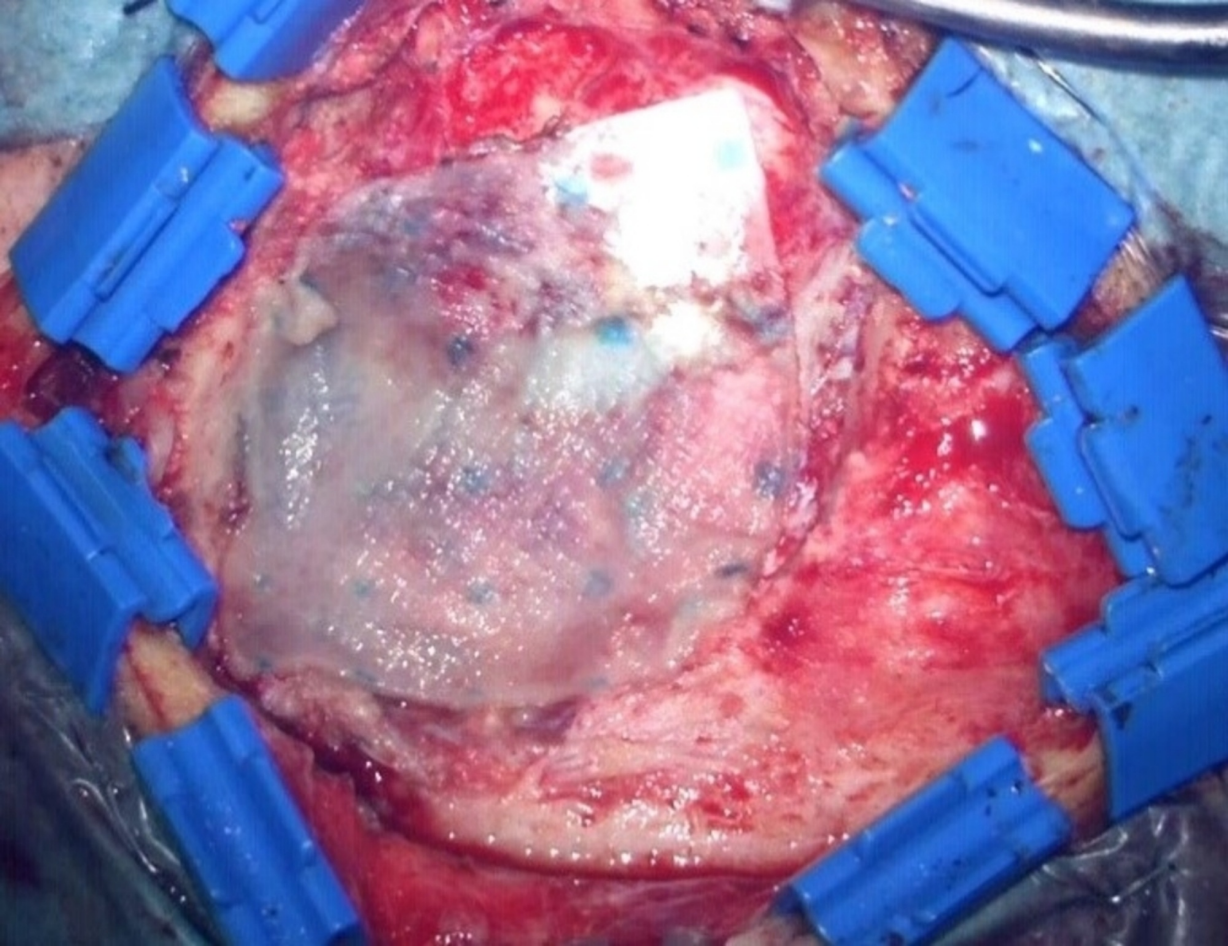
Covered with the Hemopatch

The Hemopatch was applied strictly according to the manufacturer´s recommendation (Figures 4-6): the matrix was applied dry to the dural surface, and then a dry gauze pad was gently pressed onto it for 120 seconds. In some cases, a multilayer technique was used.

Any wound infection, CSF leakage, or postoperative hemorrhage resulting in surgical revision within the follow-up period was documented.

## Results

Supratentorial, infratentorial, and transsphenoidal approaches were conducted in 17, four, and one patient, respectively. Seventeen patients had tumorous lesions; among these were four glioblastomas, three astrocytomas, one chordoid glioma, two acoustic neuromas, two pituitary adenomas, three meningiomas, one metastasis, one colloid cyst of the foramen of Monro, and five vascular lesions (three aneurysms, one microvascular compression of the trigeminal nerve, and one spontaneous intracerebral hemorrhage).

Small gaps, no visible gaps, and large gaps were covered with Hemopatch in 11, eight, and three patients. The baseline data of the study population are presented in Table 1.

**Table 1:** Baseline Data and Results (Accuracy of Dural Closure and Complications) ACA: anterior cerebral artery; CN: cranial nerve; CPA: cerebellopontine angle; CSF: cerebrospinal fluid; f: female; m: male; MCA: middle cerebral artery; Nr: Patient Number

Nr	Sex	Age	Entity	Location	Approach	Accuracy of dural closure	Complication
1	F	57	Glioblastoma multiforme	Right temporal	Transtemporal	Small gaps	
2	F	57	Acoustic neuroma	Left CPA	Retrosigmoid	No visible gaps	
3	M	71	Metastasis	Left lateral ventricle	Transtemporal	Small gap	
4	M	55	Acoustic neuroma	Left CPA	Retrosigmoid	Small gaps	Intracebellar hemorrhage
5	M	33	Chordoid glioma	Intra/supra/parasellar	Pterional	Small gaps	
6	M	73	Meningioma	Right frontal	Convexity	Large gaps	CSF leakage
7	F	36	Aneurysm	Left MCA	Pterional	No visible gaps	
8	F	48	Astrocytoma	Septum pellucidum	Transcallosal	Small gaps	
9	F	72	Intracerebral hemorrhage	Left parietal	Transparietal	No visible gaps	
10	M	44	Pituitary adenoma	Intrasellar	Transsphenoidal	Large gaps	
11	M	15	Astrocytoma	Left lateral ventricle	Transcallosal	Small gaps	
12	M	64	Glioblastoma multiforme	Right parietal	Transparietal	No visible gaps	
13	M	54	Meningioma	Sphenoid plane	Pterional	No visible gaps	
14	M	40	Meningioma	Right CPA	Retrosigmoid	Small gaps	
15	F	73	Pituitary adenoma	Intra/parasellar	Pterional	Small gaps	
16	F	32	Colloid cyst	Foramen of Monro	Transcallosal	No visible gaps	
17	F	57	Glioblastoma multiforme	Left lateral ventricle	Transtemporal	Small gaps	Wound infection
18	M	77	Glioblastoma multiforme	Right temporal	Transtemporal	Small gaps	
19	M	42	Recurrent astrocytoma	Left frontal	Transfrontal	Large gaps	
20	F	30	Vascular compression, CN. VIII	Left CPA	Retrosigmoid	No visible gaps	
21	F	46	Aneurysm	Right ACA	Pterional	No visible gaps	
22	F	50	Aneurysm	Left ACA	Pterional	Small gaps	

For the vast majority of patients, wound healing was unaffected with no CSF leakages or fistulas. The overall revision rate during follow-up of three months was 13.6% (n = 3); the surgical revision rate due to wound healing disorders (CSF leakage and infection) was 9% (n = 2). The rate of CSF leakage (n = 1) and of wound infection (n = 1) per se was 4.5%, respectively.

In one case of a patient with dural reconstruction after the removal of a large convexity meningioma, a CSF leakage was observed. In this patient, the surgical revision was conducted for secondary reconstruction and securing of the dural defect with an additional periosteal flap.

In one patient, early wound infection (within seven days after surgery) was encountered which required re-opening of the wound and evacuation of pus. Subsequently, this patient was treated with intravenous and oral antibiotics for two weeks.

One patient suffered from remote cerebellar hemorrhage, unrelated to the dural closure. We observed no other intracerebral, subdural, epidural, or subcutaneous hemorrhage resulting in surgical revision.

## Discussion

In this small, retrospective series of consecutive patients with different kinds of neurosurgical approaches to heterogeneous intracranial lesions, the surgical revision rate was quite low. The variety of intracranial lesion in this series represents the daily routine of a dedicated academic neurosurgical center.

Primary watertight dural closure forms a mainstay in intracranial neurosurgery as revision surgery carries a decided additional risk for patients [12], often resulting in prolongation of the hospital stay, and significantly increases the possibility of further internal and surgical complications [6, 13]. The start of the adjuvant treatment has to be postponed or interrupted subsequently, especially in patients with malignant cerebral tumors and postoperative CSF leakages or wound infections, which may lead to iatrogenic neurological deterioration and impaired overall outcome.

In this small and retrospective study, the overall surgical revision rate within three months was 13.6%. In one patient, a remote cerebellar hemorrhage within the crus cerebelli occurred and led to emergency repeat craniotomy with hematoma evacuation. Obviously, this complication was unrelated to the choice of dural sealant, and thus, the surgical revision rate with respect to any wound healing disorder was 9%. According to the most recent meta-analysis of post-craniotomy complications by Kinaci et al. [14], and for a more reproducible analysis of these two most important wound healing disorders, we distributed them into non-infectious postoperative CSF leakage and wound infection without CSF leakage. In both groups, we had a rate of events of 4.5% each, which is below the reported range of CSF leakages (8.2% - 8.5%) and within the reported range of infectious disorders (1.0% - 5.6%). However, the small patient number in our series relativizes this conclusion [14].

In our opinion, the administration of easily applicable dural sealants, such as the Hemopatch, can help to decrease the risk of revision surgery due to wound infections and CSF fistulas. As a topical sealing hemostat, this polyethylene glycol-covered matrix has already been evaluated in general surgery [8, 10] and in cardiac surgery [11]. The clinical results were encouraging, and no relevant neurotoxic complications were documented in animal testing [15].

Accurate dural suturing, replacement of large defects with autologous tissue, and careful repositioning of the bone flap remain the basis of any wound closure following craniotomy. However, the application of a dural sealant does support watertightness [2-3]. Furthermore, it significantly reduces the rate of surgical site infections after neurosurgical operations [14]. In our series, we found no adverse event or allergic reaction related to the Hemopatch, except for the one patient with the wound infection, and in this case, it was not possible to eliminate entirely the contribution of the collagenous material to the wound healing disorder. Despite the two wound healing disorders in our population, we conclude that Hemopatch is a safe and feasible material to seal dural defects in cranial neurosurgery. However, large and prospective clinical trials are still warranted in order to explicitly evaluate the efficacy of the novel dural sealant, Hemopatch.

Strengths and limitations

This study represents the typical neurosurgical spectrum of an oncological and vascular center. In our opinion, one of the strengths lies in the incorporation of different approaches, including those approaches to the posterior fossa. We have not used any additional artificial or autologous material for closing dural defects, and we can provide a reasonable median follow-up of three months.

However, apart from the retrospective design, the small population, and the lack of a control group, this study has several limitations. No spinal procedures have been included, no reoperations, and, in this consecutive series, no patient with an elevated CSF pressure due to hydrocephalus internus was treated.

## Conclusions

In this small retrospective study, the use of the dural onlay, Hemopatch, was found to be effective, safe, and feasible. Further controlled and prospective trials should subsequently evaluate the efficacy of different dural sealants.

## References

[1] Hutter G, von Felten S, Sailer MH, Schulz M, Mariani L (2014). Risk factors for postoperative CSF leakage after elective craniotomy and the efficacy of fleece-bound tissue sealing against dural suturing alone: a randomized controlled trial. J Neurosurg.

[2] Kumar A, Maartens NF, Kaye AH (2003). Evaluation of the use of BioGlue in neurosurgical procedures. J Clin Neurosci.

[3] Fishman AJ, Marrinan MS, Golfinos JG, Cohen NL, Roland JT Jr (2004). Prevention and management of cerebrospinal fluid leak following vestibular schwannoma surgery. Laryngoscope.

[4] Samii M, Matthies C (1997). Management of 1000 vestibular schwannomas (acoustic neuromas): the facial nerve--preservation and restitution of function. Neurosurgery.

[5] Fang C, Zhu T, Zhang P, Xia L, Sun C (2017). Risk factors of neurosurgical site infection after craniotomy: a systematic review and meta-analysis. Am J Infect Control.

[6] Grotenhuis JA (2005). Costs of postoperative cerebrospinal fluid leakage: 1-year, retrospective analysis of 412 consecutive nontrauma cases. Surg Neurol.

[7] Lewis KM, Kuntze CE, Gulle H (2015). Control of bleeding in surgical procedures: critical appraisal of Hemopatch (sealing hemostat). Med Devices (Auckl).

[8] Fingerhut A, Uranues S, Ettorre GM (2014). European initial hands-on experience with Hemopatch, a novel sealing hemostatic patch: application in general, gastrointestinal, biliopancreatic, cardiac, and urologic surgery. Surg Technol Int.

[9] Ikeme S, Weltert L, Lewis KM (2018). Cost-effectiveness analysis of a sealing hemostat patch (Hemopatch) vs standard of care in cardiac surgery. J Med Econ.

[10] Ulrich F, Ettorre GM, Weltert L, Oberhoffer M, Kreuwel H, De Santis R, Kuntze E (2016). Intra-operative use of Hemopatch(R): interim results of a nationwide European survey of surgeons. Surg Technol Int.

[11] Weltert L, D'Aleo S, Chirichilli I, Falco M, Turani F, Bellisario A, De Paulis R (2016). Prospective randomized clinical trial of Hemopatch topical sealant in cardiac surgery. Surg Technol Int.

[12] Giovanni S, Della Pepa GM, La Rocca G, Lofrese G, Albanese A, Maria G, Marchese E (2014). Galea-pericranium dural closure: can we safely avoid sealants?. Clin Neurol Neurosurg.

[13] Horowitz G, Fliss DM, Margalit N, Wasserzug O, Gil Z (2011). Association between cerebrospinal fluid leak and meningitis after skull base surgery. Otolaryngol Head Neck Surg.

[14] Kinaci A, Algra A, Heuts S, O'Donnell D, van der Zwan A, van Doormaal T (2018). Effectiveness of dural sealants in prevention of cerebrospinal fluid leakage after craniotomy: a systematic review. World Neurosurg.

[15] Lewis KM, Sweet J, Wilson ST, Rousselle S, Gulle H, Baumgartner B (2118). Safety and efficacy of a novel, self-adhering dural substitute in a canine supratentorial durotomy model. Neurosurgery.

